# Exosomes/microvesicles target SARS-CoV-2 via innate and RNA-induced immunity with PIWI-piRNA system

**DOI:** 10.26508/lsa.202101240

**Published:** 2021-12-03

**Authors:** Shoeb Ikhlas, Afia Usman, Dongkyeong Kim, Dongsheng Cai

**Affiliations:** 1 Department of Molecular Pharmacology, Albert Einstein College of Medicine, New York City, NY, USA; 2 Institute for Neuroimmunology and Inflammation, Albert Einstein College of Medicine, New York City, NY, USA

## Abstract

Murine neural stem cells (NSCs) were recently shown to release piRNA-containing exosomes/microvesicles (Ex/Mv) for exerting antiviral immunity, but it remains unknown if these Ex/Mv could target SARS-CoV-2 and whether the PIWI-piRNA system is important for these antiviral actions. Here, using in vitro infection models, we show that hypothalamic NSCs (htNSCs) Ex/Mv provided an innate immunity protection against SARS-CoV-2. Importantly, enhanced antiviral actions were achieved by using induced Ex/Mv that were derived from induced htNSCs through twice being exposed to several RNA fragments of SARS-CoV-2 genome, a process that was designed not to involve protein translation of these RNA fragments. The increased antiviral effects of these induced Ex/Mv were associated with increased expression of piRNA species some of which could predictably target SARS-CoV-2 genome. Knockout of piRNA-interacting protein PIWIL2 in htNSCs led to reductions in both innate and induced antiviral effects of Ex/Mv in targeting SARS-CoV-2. Taken together, this study demonstrates a case suggesting Ex/Mv from certain cell types have innate and adaptive immunity against SARS-CoV-2, and the PIWI-piRNA system is important for these antiviral actions.

## Abstract (precis)

Murine neural stem cell exosomes/microvesicles can work to reduce SARS-CoV-2, an effect that can be adaptively enhanced via viral RNA fragment stimulation, which requires the PIWI-piRNA system.

## Introduction

Murine neural stem cells (NSCs), in particular hypothalamic NSCs (htNSCs) (1, 2, 3, 4, 5, 6), are known to abundantly release exosomes/microvesicles (Ex/Mv) that are enriched with small RNAs such as miRNAs (3, 4). Recently, we revealed that murine NSC Ex/Mv comprise both innate and adaptive immunity effects in breaking pseudotyped SARS-CoV-2 and HIV-based recombinant lentivirus (2). Also, as demonstrated in this study (2), NSC Ex/Mv contain a vast variety of P-element induced wimpy testis (PIWI)–interacting RNA (piRNA) species, a type of noncoding small RNAs which have been related to host–pathogen interactions in insects (7, 8, 9, 10, 11, 12, 13). As established, piRNAs are often composed of 24–32 nucleotides, slightly larger than miRNAs (21–24 nucleotides). Compared with miRNAs, piRNAs have many levels of distinct features, for example, piRNAs do not have a conserved sequence and do not depend on Dicer machinery in biogenesis (14, 15). Furthermore, piRNAs induce genic and intergenic silencing such as transposon silencing and splicing (14, 15, 16), a function which manifests an immunity-like action against transposon invasion. Mammalian piRNAs are known to be produced mainly in the reproductive tissue (15, 17, 18, 19) and the neural tissue (20, 21, 22, 23, 24). The biological functions of piRNAs importantly require PIWI proteins, including PIWI-like proteins PIWIL1 and PIWIL2 in mammals, whereas PIWIL2 appears to be particularly relevant in the brain (2, 21). Herein, using in vitro models of SARS-CoV-2 infection, we studied if murine NSC Ex/Mv could have an innate immunity action against this pandemic virus, whether adaptive immunity response could be developed to enhance the antiviral action of NSC Ex/Mv, and if the PIWIL2-piRNA system could be involved in the antiviral immunity of NSC Ex/Mv.

## Results

### Antiviral effects of htNSC Ex/Mv against SARS-CoV-2 infection

**Figure 1.**
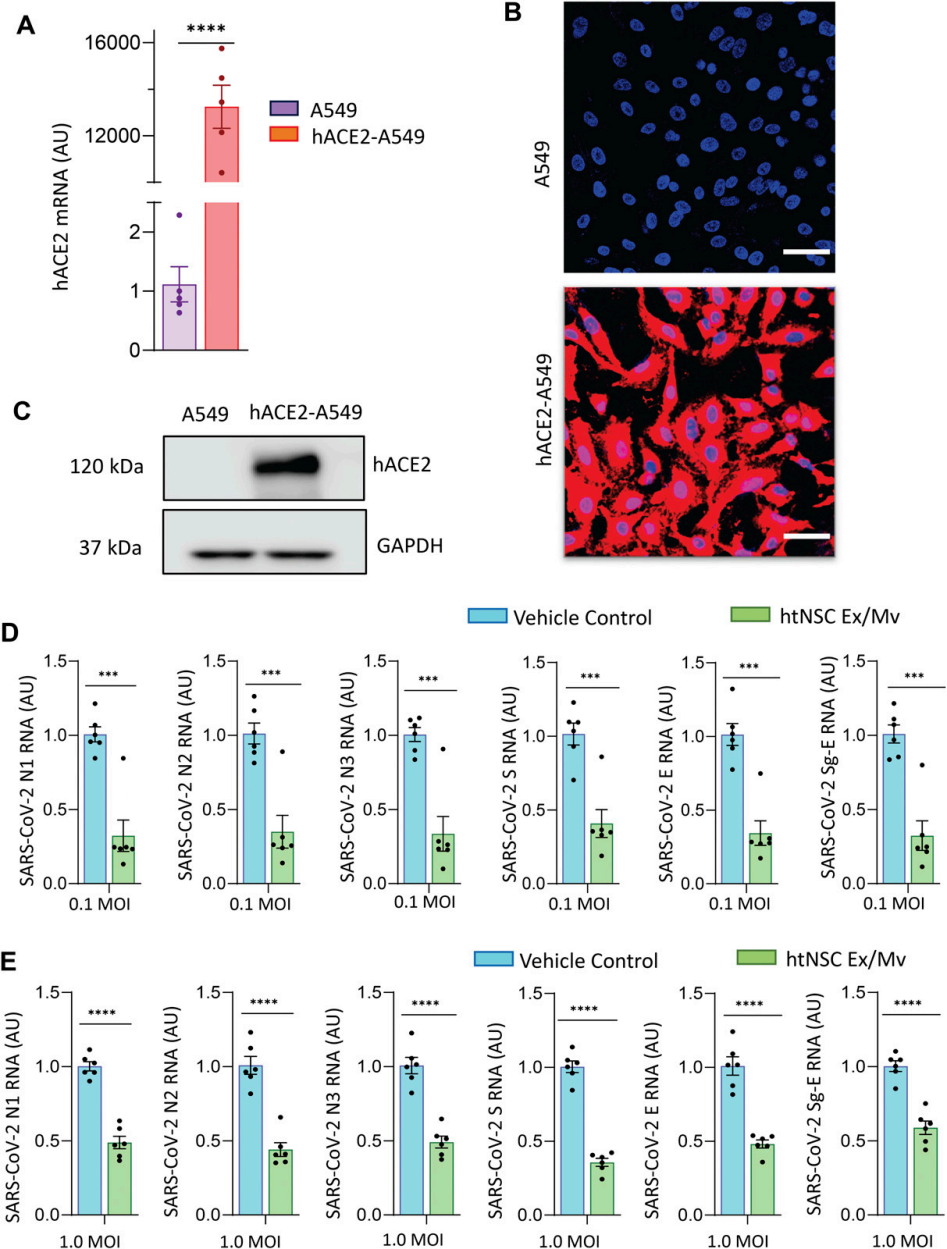
Antiviral effects of htNSC Ex/Mv against SARS-CoV-2. **(A, B, C)** hACE2-A549 cells were evaluated for (A) hACE2 mRNA via qPCR, (B) hACE2 immunostaining, and (C) hACE2 protein via Western blot. **(A)** hACE2 mRNA levels in hACE2-A549 cells as fold change compared with the levels in A549 cells of which the average was adjusted as 1. AU, arbitrary unit. **(B)** hACE2 staining is shown in red, whereas DAPI staining in blue reveals nuclei of all cells in slides. Scale bar, 50 μm. **(C)** Blotting for GAPDH in the same membrane was used as a technical control. **(D, E)** hACE2-A549 cells were infected with (D) 0.1 MOI and (E) 1.0 MOI of SARS-CoV-2 virus USA-WA1/2020 and treated with htNSC Ex/Mv (3.5 μg per 200,000 cells) or vehicle control, and 2 d later these cells were lysed for measuring the levels of N1, N2, N3, S, and E segments as well as sub-genomic E region (Sg-E) of SARS-CoV-2 RNA genome via qPCR. Data are expressed as fold changes with respect to control group of which the average was adjusted as one. ***P < 0.001, ****P < 0.0001, two-tailed unpaired *t* test was applied between groups as indicated (A, D, E); data reflect mean ± SEM.

Ex/Mv that are released from mouse NSCs, including htNSC, were recently found to reduce infection of several experimentally recombinant RNA viruses (2). In this context, this project was to further investigate if these Ex/Mv could inhibit SARS-CoV-2 infection. We used human alveolar basal epithelial cell line A549, a cell model that has been used to study SARS-CoV-2 infection (25, 26, 27). Although A549 cells contain human angiotensin-converting enzyme 2 (hACE2) which is known to facilitate the entry of SARS-CoV-2 into cells, our pilot experiment revealed that SARS-CoV-2 infection in A549 cells was relatively modest, most likely due to low expression level of hACE2 on the surface of A549 cells. Therefore, through lentiviral hACE2 induction and cell selection, we developed an A549 cell line which stably overexpressed hACE2, namely, hACE2-A549 cells. As shown in Fig 1A, hACE2 mRNA levels dramatically increased in hACE2-A549 cells compared with A549 cells. Using immunostaining (Fig 1B) and Western blotting (Fig 1C), we further confirmed that hACE2 was strongly expressed in hACE2-A549 cells, and compared with these heavy levels of experimental induction, its endogenous expression in A549 cells was barely appreciable. We thus used this in vitro system to study if NSC Ex/Mv could provide any therapeutic effect against SARS-CoV-2 infection.

SARS-CoV-2 USA-WA1/2020 viruses were generated and quantitated for MOI according to the method as recently published (28). MOI 0.1 of SARS-CoV-2 which represents a regular dose of infection has been used in research (29, 30, 31). We included regular dose MOI 0.1 as well as high-dose MOI 1.0 of SARS-CoV-2 in our study, so that the conditions of high-level infection were also assessed. We generated and purified Ex/Mv from cultured mouse htNSCs, as performed previously (2). Cultured hACE2-A549 cells at an appropriate density were added with MOI 0.1 or 1.0 of SARS-CoV-2 USA-WA1/2020 and treated with or without htNSC-derived Ex/Mv. The dose of Ex/Mv was 3.5 μg for 200,000 hACE2-A549 cells, which was chosen based on our pilot experiment. After 2 d of incubation, hACE2-A549 cells were lysed and analyzed for the quantity of SARS-CoV-2 RNA genomes through quantitative PCR (qPCR). We used five different qPCR conditions to cover five different regions of SARS-CoV-2 genomic RNA, including different N, S, and E regions which are responsible for encoding nucleocapsid, spike, and envelop proteins, respectively. We found that treatment with htNSC Ex/Mv provided a significant effect against SARS-CoV-2 under both infection dose conditions (Fig 1D and E). When infection dose was 10 times higher (1.0 MOI), the antiviral effect of htNSC Ex/Mv was still significant, although to a slightly less extent compared with the effect against regular dose (MOI 0.1) infection. Furthermore, we used qPCR to examine the levels of viral sub-genomic E region (Sg-E) which has been established to indicate viral replication in infected cells (32). As shown in Fig 1D and E, we found that treatment of htNSC Ex/Mv led to significant reductions in the expression levels in Sg-E under both MOI 0.1 and MOI 1.0 infection conditions. Thus, htNSC Ex/Mv have an innate immunity ability to reduce SARS-CoV-2, including an effect in suppressing viral replication in infected cells.

### Reduction of htNSC Ex/Mv piRNAs by PIWIL2 knockout

According to piRNA database deposition, the numbers of piRNA species are huge, and a mouse contains a very big number of different piRNA species (33, 34). Based on this information, we recently analyzed piRNA species with sequences that could potentially target SARS-CoV-2 RNA genome (2). Our analysis covered different segments of SARS-CoV-2 genome, including the sequences that encode spike protein (S); envelope protein (N); membrane protein (M); and nucleocapsid protein (N); open reading frame (Orf) sequences Orf1ab, 3a, 6, 7a, 7b, 8, and 10; untranslated region (UTR) sequences at 5′ end and 3′ end; and gap sequences between some of these segments. As we have reported (2), this search led to identification of a list of piRNAs some of which possibly target SARS-CoV-2 genome, and we further confirmed that quite some of these piRNAs were detectable in NSC Ex/Mv. In this context and given the effect of NSC Ex/Mv against SARS-CoV-2 as demonstrated above, we went on to study if the piRNA system could contribute to the antiviral effects of NSC Ex/Mv. Because it was experimentally unfeasible to directly target individual piRNAs, we developed a strategy by targeting PIWI protein which is required for biogenesis and function of piRNAs. Four PIWI homologs (PIWIL1-4) are known to exist in mammals, but only three (PIWIL1, 2, and 4) were found in mice. Research revealed that PIWI proteins are present in the nervous system (20), and we recently confirmed that PIWIL2 was significantly present in several types of NSCs, including htNSCs (2). We decided to study if loss of PIWIL2 could affect the antiviral effects of htNSC Ex/Mv, using SARS-CoV-2 infection model as established above.

**Figure 2.**
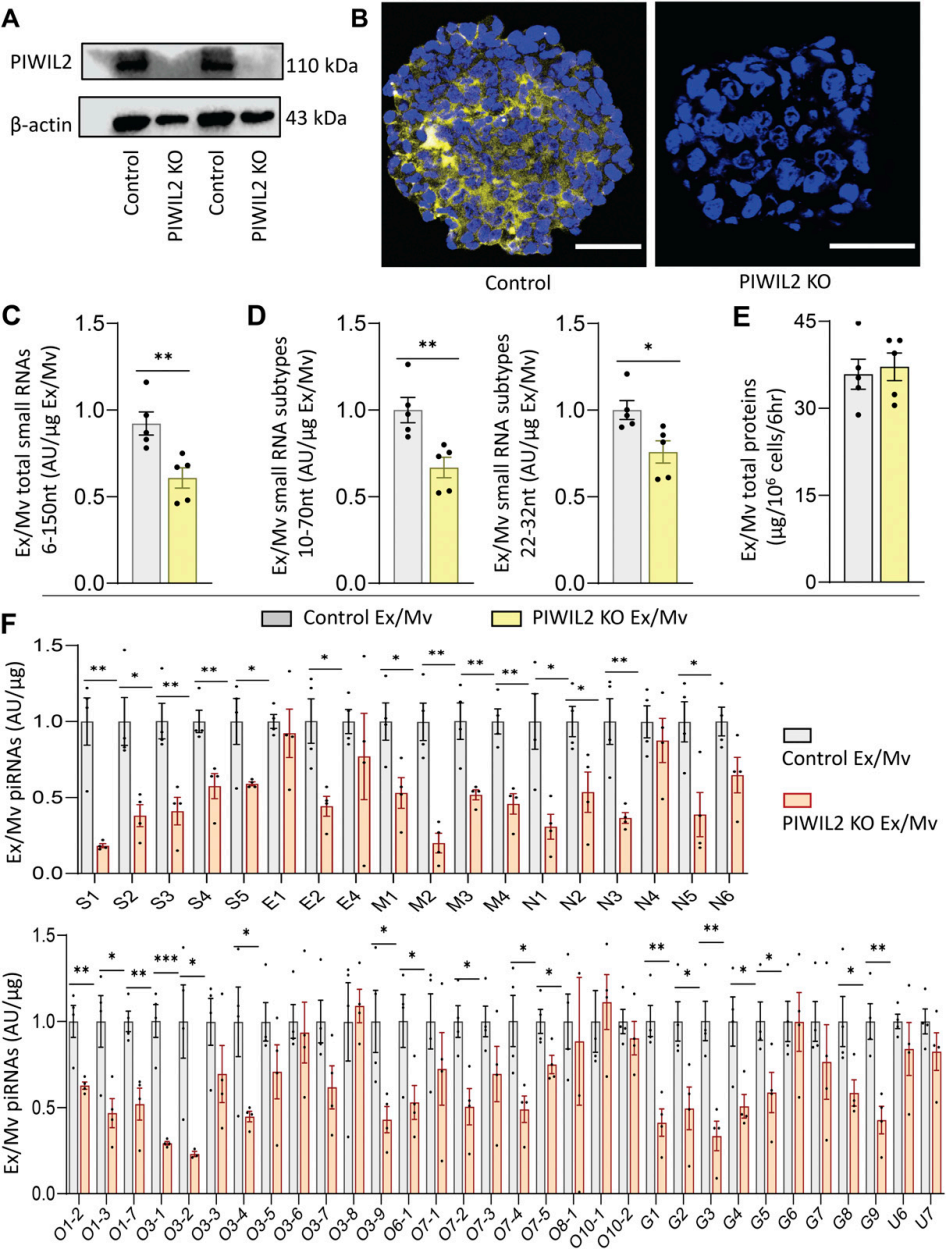
Piwil2 knockout in htNSCs leading to decreased Ex/Mv piRNAs. **(A, B)** Knockout of Piwil2 gene in htNSCs was done using mammalian CRISPR lentiviral transduction followed by blasticidin selection to create stable htNSC-PIWIL2 KO cell lines. These htNSC-PIWIL2 KO cells were evaluated in comparison with control htNSCs using (A) Western blot for Piwil2 protein with β-actin as a technical control, and (B) Piwil2 immunostaining (yellow) of neurospheres, whereas DAPI nuclear staining (blue) was used as a reference. Scale bars, 50 μm. **(C, D, E, F)** Equal amount of Ex/Mv from htNSC-PIWIL2 KO (labelled as PIWIL2 KO Ex/Mv) versus control htNSCs (labelled as Control Ex/Mv) were analyzed for (C) total small RNAs at the size of 6–150 nt, including (D) small RNA subpopulations at the size of 10–70 nt or 22–32 nt via small RNA analysis assay using 2100 bioanalyzer, (E) total protein levels via BCA, and (F) a list of piRNAs via qPCR. AU, arbitrary unit. **(F)** Labels of piRNA species corresponded to a region in SARS-CoV-2 genome including regions for making spike protein (S), membrane protein (M) and nucleocapsid (N), open reading frames (O), UTR (U), and gap sequences which link some of these regions (G). Please refer to Table 1 for the labels of these piRNA species. Data of each piRNA is expressed as fold changes relative to the average values of control adjusted as 1. *P < 0.05, **P < 0.01, ***P < 0.001, two-tailed unpaired *t* test was applied between groups as indicated, data reflect mean ± SEM.

Using CRISPR/Cas9 knockout technology, we deleted a genomic sequence for encoding PIWIL2 in htNSCs, leading to the establishment of htNSC-PIWIL2 KO cell line. As verified through Western blot in Fig 2A, PIWIL2 protein was absent in htNSC-PIWIL2 KO, whereas it was expressed in control htNSCs. Immunostaining further confirmed that PIWIL2 protein was absent in htNSC-PIWIL2 KO neurospheres, compared with its presence in control htNSC neurospheres (Fig 2B). We then analyzed Ex/Mv from cultured htNSC-PIWIL2 KO compared with that from control htNSCs through small RNA bioanalyzer assay. As shown in Fig 2C, PIWIL2 knockout led to a great reduction in total small RNAs with size less than 150 nucleotides (nt). We further examined Ex/Mv small RNA subpopulations for the size range of 10–70 nt and 22–32 nt, and the results confirmed these levels of reductions in htNSC-PIWIL2 KO Ex/Mv compared with control htNSC Ex/Mv (Fig 2D). Of note, total Ex/Mv protein levels did not significantly differ between htNSC-PIWIL2 KO-derived Ex/Mv and control htNSC-derived Ex/Mv (Fig 2E). Certainly, the total protein levels do not necessarily reflect individual proteins, so protein constituents in Ex/Mv could still be changed; regardless, it was clear to us that PIWIL2 ablation led to substantial reductions in Ex/Mv total small RNAs more than total proteins. We further analyzed individual piRNAs for expression levels in Ex/Mv from htNSC-PIWIL2 KO compared with Ex/Mv from control htNSCs. As we previously profiled (2), mouse piRNA library contains a collection of piRNAs which could match against the sense or antisense sequence of SARS-CoV-2 RNA genome according to the criteria from *Caenorhabditis elegans* research. In this study, we narrowed down to a list of these piRNAs which were relatively detectable in htNSC Ex/Mv, as summarized in Table 1. We found that most of these piRNAs decreased their expression levels in in Ex/Mv from htNSC-PIWIL2 KO compared with Ex/Mv from control htNSCs (Fig 2F). Hence, we generated htNSC Ex/Mv in which piRNAs were reduced through genetic ablation of PIWIL2 in htNSCs.

**Table 1.** The piRNA sequences and labels in this study.

piRNA ID (piRNAQuest)	Sequence	Label
mmu_piRNA_553635	TGAAGAAGAGCAAGAAGGAAAGTGAA	O1-2
mmu_piRNA_443751	TATTTAAACTGTCTTATGTGTCTCCA	O1-3
mmu_piRNA_923720	TTTCCAGAGTTGTTGTACCAATTTCCAAT	O1-7
mmu_piRNA_555154	TGAAGACCCAGTCCCTACCTTAGCCTA	S1
mmu_piRNA_801725	TGTGAAGGTGTCTTTGTCACTAATAGATG	S2
mmu_piRNA_562740	TGAAGTCTGCCTGTGAAGTCTGCCTGTGA	S3
mmu_piRNA_498273	TCCTGAAGAAGAATCACAATCGTTCACAGT	S4
mmu_piRNA_233057	GTGAAGTCTGCCTGTGAAGTCTGCCT	S5
mmu_piRNA_858506	TTCAAGGCCAGCAGCTACAGAGTGAG	O3-1
mmu_piRNA_561925	TGAAGTAACTGTGTATACTGGGTATA	O3-2
mmu_piRNA_443463	TATTGTGTGAATTTGGTTTTGTGGTG	O3-3
mmu_piRNA_443462	TATTGTGTGAATTTGGTTTTGTCATT	O3-4
mmu_piRNA_443460	TATTGTGTGAATTTGGTTTTGTCAGG	O3-5
mmu_piRNA_88635	ATTGTGTGAATTTGGTTTTGTCCTGG	O3-6
mmu_piRNA_88634	ATTGTGTGAATTTGGTTTTGTCATGG	O3-7
mmu_piRNA_88633	ATTGTGTGAATTTGGTTGTGTCATGG	O3-8
mmu_piRNA_86326	ATGTTCTTCAGGCTCCCCTGCAGGTTTGTTTTTG	O3-9
mmu_piRNA_104250	CAGAAGATCAGGAACTAACAGGCAAA	E1
mmu_piRNA_181390	GAAGGTTTTACAAGATAAGGGGCTTC	E2
mmu_piRNA_466370	TCAGGACCTCTAGAAGAACAATCAGT	E4
mmu_piRNA_419319	TAGTTTTTCTGTTCAATGGTTCATGA	G1
mmu_piRNA_419318	TAGTTTTTCTGTTAAGTGAAGAGGGG	G2
mmu_piRNA_865863	TTCCAAACAGAAAATGCAGCTTTCGA	G3
mmu_piRNA_475430	TCCAAACAGAAGAACTAGCAAAGCAA	G4
mmu_piRNA_475429	TCCAAACAGAAAAGCTTAAAGTTAAG	G5
mmu_piRNA_700126	TGCTTCTTTCAGACTTCCCTTCTGTCT	M1
mmu_piRNA_379382	TAGCAATTCCACCGGTGGAAACAGTA	M2
mmu_piRNA_167046	CTGTACAAGCAAAGCTCTTGGGAGGT	M3
mmu_piRNA_142622	CCTGTATGCAGCAAAATGTTGGGTCC	M4
mmu_piRNA_432244	TATGAGGACTTTGAAAGTTGGACTAA	O6-1
mmu_piRNA_25718	AATTTGCTTTTGCTTTAATCCCAGGT	O7-1
mmu_piRNA_867226	TTCCAGAAGAGCCAGGTTCAGTTCCC	O7-2
mmu_piRNA_320536	TACACTCTTGGTAGTGGGGAGCCATGGGATC	O7-3
mmu_piRNA_934618	TTTTAGCCTTTCTGCCGTTCTGACA	G6
mmu_piRNA_292200	TAAGGAATAGCAGAATGCTTTAATGC	G7
mmu_piRNA_934618	TTTTAGCCTTTCTGCCGTTCTGACA	O7-4
mmu_piRNA_292200	TAAGGAATAGCAGAATGCTTTAATGC	O7-5
mmu_piRNA_658285	TGCAGCTACAGTTGTGTGCTACTCTC	O8-1
mmu_piRNA_718264	TGGACTTCCCTATGGTCGTGACCTTTCCCGCC	N1
mmu_piRNA_153125	CTCCATGAGCAGTGCTGGGAACAGTAGCAGGAAC	N2
mmu_piRNA_288153	TAAGATGGTATTTCTAGCTGTTAGGT	N3
mmu_piRNA_312079	TAATTTCCTTGGGTTTGTTTTTGGTC	N4
mmu_piRNA_657627	TGCAGCAGATTTCTTATTTGGGTTTT	N5
mmu_piRNA_521705	TCTGCAGCAGGAAGAGTCTTATTGTCC	N6
mmu_piRNA_647225	TGCAAACCACACAAGGCTTTATTCCG	G8
mmu_piRNA_679242	TGCCTTGTGTGGTGAAGGGTCTGCAC	G9
mmu_piRNA_865467	TTCATTCTGCACAATGTTATTCCTGTGAGG	O10-1
mmu_piRNA_811305	TGTGCTATGTAGTTCTGACTGGTGGA	O10-2
mmu_piRNA_402005	TAGGGAGAGCTGCCCCTCCAGTTGTCTGT	U6
mmu_piRNA_43308	AGAAAAAGTGGTGGCTCTTTTGAAGG	U7

The piRNA sequences are presented using T in place of U. Labels are named based on analyzing SARS-CoV-2 genome as similarly did previously (2).

### Reduced effects by piRNA-impaired htNSC Ex/Mv against coronaviruses

**Figure 3.**
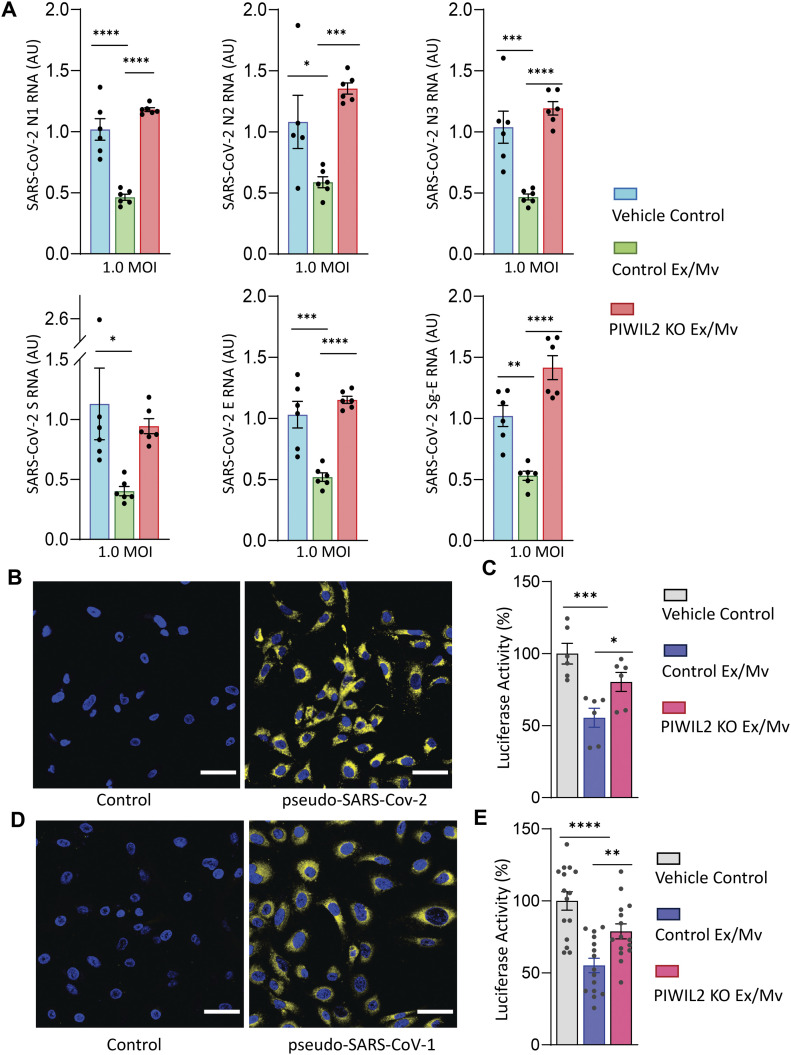
Loss of antiviral effects by htNSC Ex/Mv due to Piwil2 knockout. **(A)** hACE2-A549 cells were infected with 1.0 MOI SARS-CoV-2 virus and treated with Ex/Mv (3.5 μg per 200,000 cells) isolated from htNSC PIWIL2 KO (labelled as “PIWIL2 KO Ex/Mv”) versus control htNSCs (labelled as “Control Ex/Mv”) or vehicle control (labelled as “Vehicle Control”) for 2 d. These cells were harvested and analyzed for N1, N2, N3, S, and E gene segments as well as sub-genomic E region (Sg-E) of SARS-CoV-2. Data are represented as fold change with respect to control whose average was adjusted as one. **(B, C, D, E)** Immunostaining for viral luciferase was used to confirm the infection models of (B) pseudotyped SARS-CoV-2 and (D) pseudotyped SARS-CoV-1 in hACE2-A549 cells, and using these infection models, hACE2-A549 cells were infected with (C) pseudotyped SARS-CoV-2 or (E) pseudotyped SARS-CoV-1 and were treated with the same amount of Ex/Mv (0.7 μg per 40,000 cells) from htNSC PIWIL2 KO versus Control htNSCs for 3 d. These cells were then harvested for measuring luciferase activities to quantitatively report the infection levels of these pseudotyped viruses. **(B, D)** Immunostaining for uninfected hACE2-A549 cells were included as control references (B, D). *P < 0.05, **P < 0.01, ***P < 0.001, ****P < 0.0001, one-way ANOVA followed by Tukey’s post hoc test; data reflect mean ± SEM.

We subsequently examined if htNSC Ex/Mv with reduced piRNAs could still maintain the effect against SARS-CoV-2. To do so, we continued to use SARS-CoV-2 USA-WA1/2020, as established in Fig 1. Cultured hACE2-A549 cells at an appropriate density were infected with MOI 1.0 SARS-CoV-2 and treated with the same amount of Ex/Mv (3.5 μg Ex/Mv for 200,000 hACE2-A549 cells) from htNSC-PIWIL2 KO versus control htNSCs. 2 d later, hACE2-A549 cells were harvested and lysed for measuring SARS-CoV-2 RNA genomic copies. We found that, whereas Ex/Mv from control htNSCs significantly reduced SARS-CoV-2 infection, the same amount of Ex/Mv from htNSC-PIWIL2 KO did not provide this antiviral effect (Fig 3A). We further used sub-genomic E region qPCR to evaluate how viral replication could be affected. As shown in Fig 3A, treatment with control htNSC Ex/Mv led to reduction in SARS-CoV-2 replication, but treatment with htNSC-PIWIL2 KO Ex/Mv failed to do so. Thus, the PIWIL2-piRNA system is important for the antiviral effect of htNSC Ex/Mv in targeting SARS-CoV-2.

Since PIWI-dependent piRNAs are widely ranged, we predicted that the potential piRNA antiviral mechanism of NSC Ex/Mv in targeting an RNA virus should not be limited to SARS-CoV-2 genome. Indeed, we recently reported that NSC Ex/Mv had antiviral effects against a model of pseudotyped SARS-CoV-2 based on the genome of glycoprotein-deficient vesicular stomatitis virus (ΔG-VSV), and we identified a list of mouse piRNAs with sequences putatively against ΔG-VSV genomic backbone (2). Here, given that we have developed htNSC-PIWIL2 KO model, we designed to test if the PIWIL2-piRNA system could be necessary for the effect of NSC Ex/Mv against ΔG-VSV-based pseudotyped virus. To align with this study, we used hACE2-A549 cell infection model and confirmed that pseudotyped SARS-CoV-2 (10^5^ pfu/10^6^ cells) efficiently infected these cells, as reveled by immunostaining for luciferase in ΔG-VSV (Fig 3B). Using this infection model, we treated these cells with the same amount of Ex/Mv (0.7 μg Ex/Mv for 40,000 hACE2-A549 cells) derived from htNSC-PIWIL2 KO versus control htNSCs for 3 d. Then, these cells were lysed and measured for luciferase activity to reflect the infection levels. As shown in Fig 3C, Ex/Mv from control htNSCs significantly inhibited the infection, but ablation of PIWIL2 led to a significant reduction in this antiviral effect.

For comparison, we also used the pseudotyped system to examine SARS-CoV-1 because it uses a different spike protein for infection. We generated this pseudotyped virus by incorporating SARS-CoV-1 spike protein into ΔG-VSV. Compared with pseudotyped SARS-CoV-2, pseudotyped SARS-CoV-1 had more difficulty and variations in infecting hACE2-A549 cells (Fig 3D), thus we decided to increase biological replicates for sufficient statistical power. Using this infection model with enough biological replicates, we provided the treatment with the same amount of Ex/Mv (0.7 μg for 40,000 hACE2-A549 cells) derived from htNSC-PIWIL2 KO versus control htNSCs for 3 d. As shown in Fig 3E, luciferase activity measurement revealed that htNSC Ex/Mv clearly had a significant antiviral action, but this effect was reduced when PIWIL2 was ablated. Taken together, all these data support that the PIWIL2-piRNA system is important for htNSC Ex/Mv for the effects against multiple types of viruses.

### Induced htNSC Ex/Mv piRNAs through viral RNA fragment stimulation

**Figure 4.**
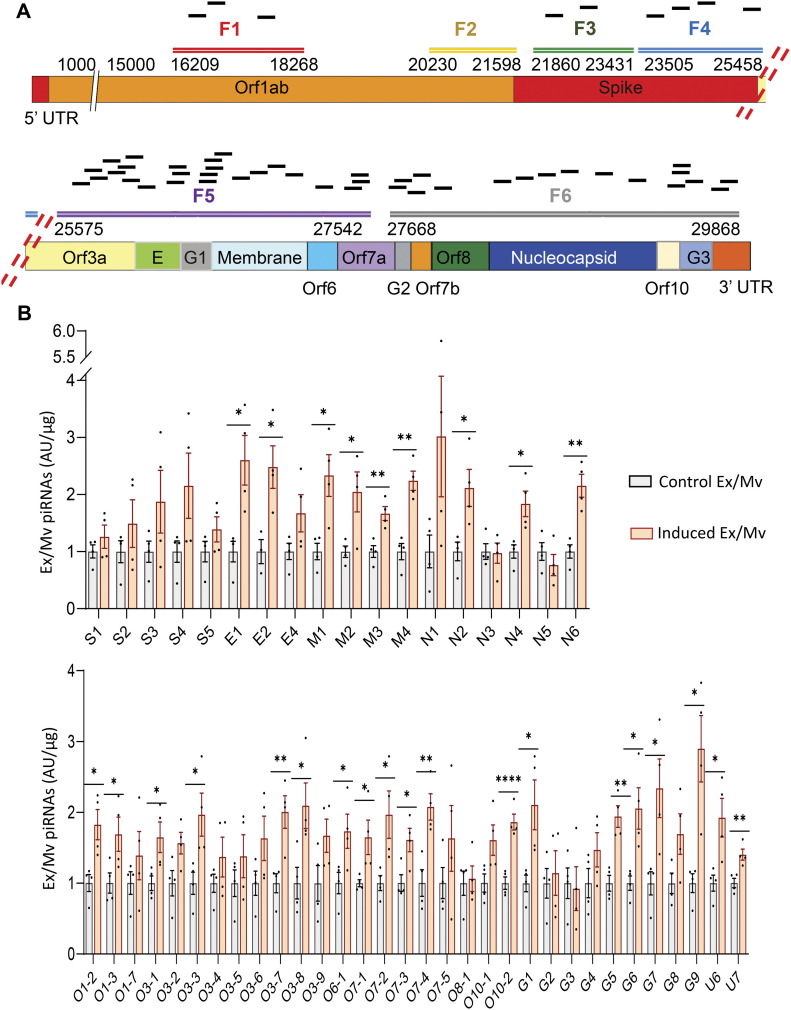
Induced Ex/Mv in htNSCs via SARS-CoV-2 RNA fragment stimulation. **(A)** Schematic of SARS-CoV-2 genomic fragments (F1-F6) which were cloned into pCR-Blunt II-TOPO vector leading to productions of F1-6 RNA fragments via in vitro transcription. Small black bars above F1-6 indicate piRNA species with the sequences that correspond to the fragment below. **(B)** htNSCs were transfected with a mixture of F1-6 RNA fragments, this process was repeated 2 wk later, and these cells were established as induced htNSCs. Small RNAs were extracted from an equal amount of Ex/Mv from induced htNSCs (labelled as induced Ex/Mv) versus control htNSCs (labelled as Control Ex/Mv) and measured for piRNAs which could potentially target SARS-CoV-2 genome. Data of each piRNA in induced htNSC Ex/Mv are expressed as fold changes relative to the average values of control htNSC Ex/Mv adjusted as one. Please refer to Table 1 regarding labels of these piRNA species. *P < 0.05, **P < 0.01, ***P < 0.001, ****P < 0.0001, two-tailed unpaired *t* test was applied between groups as indicated; data reflect mean ± SEM.

In our previous work, we showed that exposure to pseudotyped viruses to mouse NSCs led to increased Ex/Mv production of some piRNA species whose sequences could match against RNA genomes of these viruses according to the criteria from *C. elegans* research, suggesting that piRNA-based adaptive immunity could exist (2). In this context, we decided to study if exposure of RNA fragments of SARS-CoV-2 genome to NSCs could lead to an enhanced Ex/Mv production of some piRNAs which could potentially target this viral RNA genome. As elucidated in Fig 4A, we designed six RNA fragments, namely, F1–6, which corresponded to a fragment of encoding sequence for spike (S), membrane (M), nucleocapsid (N), or envelop (E) protein, and/or a fragment of open reading frame including Orf1ab, Orf3a, Orf6, Orf7a, Orf7b, Orf8 and Orf10, 3′ untranslated terminal region (3′ UTR) or gap sequences (G1-3) in the SARS-CoV-2 genome. F1–6 were designed to cover the piRNA species in Table 1, and the relative position of these piRNAs are elucidated in Fig 4A. There are still additional piRNAs that correspond to F1-6 sequences, which are not displayed in Fig 4A or Table 1, since we have not analyzed them due to the capacity of the experiments. Using SARS-CoV-2 genome as the template, we produced these RNA fragments through an in vitro transcription system and then purified and quantified each of these fragments. Subsequently, htNSCs were exposed to the mixture of F1–6 through transfection, and 2 wk later, a second exposure of F1–6 was introduced to these cells through transfection. F1–6 RNA fragments did not contain elements for protein translation, so the experiment did not involve protein synthesis from these sequences. After two exposures with F1–6, these induced htNSCs were maintained and expanded in culture for at least three to four generations before Ex/Mv were collected from these cells. We isolated small RNAs from Ex/Mv that were released by these induced htNSCs versus control htNSCs for piRNA qPCR. Compared with htNSC Ex/Mv, induced htNSC Ex/Mv showed increased levels in many of these piRNAs which corresponded to F1–6 sequences. Among 50 piRNA species in this qPCR assay, 28 of them showed significant increases in induced htNSC Ex/Mv compared with htNSC Ex/Mv (Fig 4B). Among these 28 piRNA species, some of them matched against the sequences of F1-6, whereas others surprisingly matched against the complementary sequences of F1–6, suggesting that RNA stimulation might use multiple different processes to induce piRNA biogenesis. Altogether, stimulation of htNSCs with SARS-CoV-2 RNA fragments can lead to increased production of some sequence-matched piRNAs and assemble them into Ex/Mv for release.

### Enhanced antiviral action of induced htNSC Ex/Mv for SARS-CoV-2

**Figure 5.**
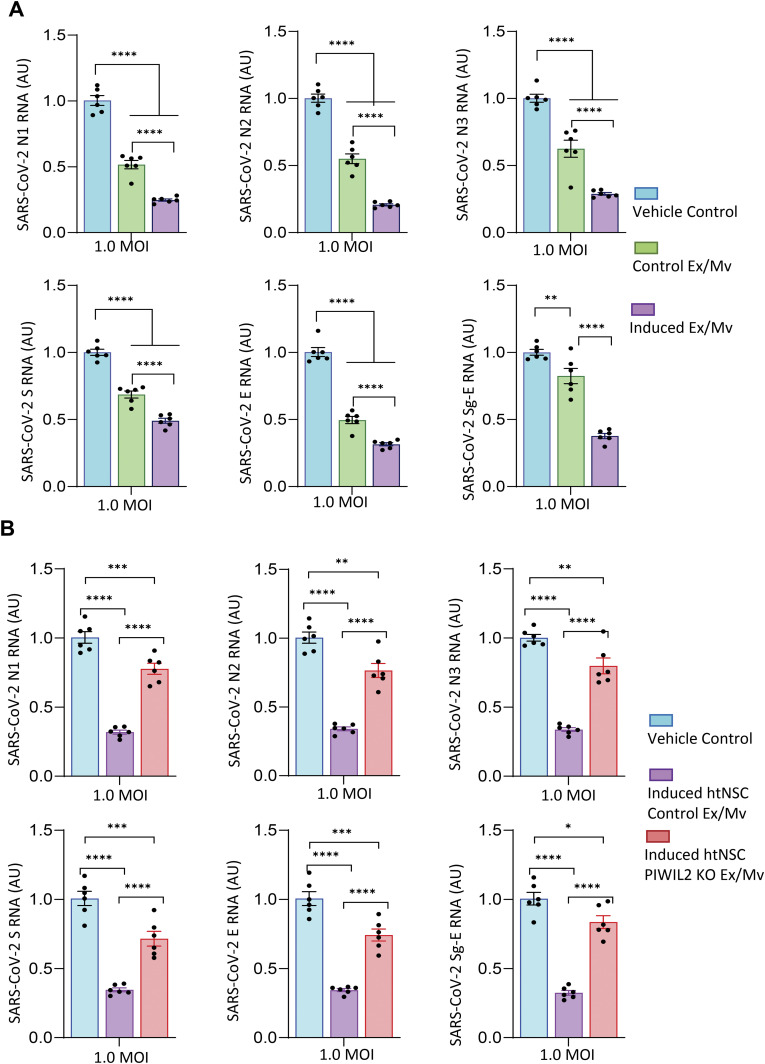
Piwil2-dependent enhanced antiviral effects of induced htNSC Ex/Mv. **(A)** hACE2-A549 cells were infected with SARS-CoV-2 and treated with the same amount of Ex/Mv (3.5 μg per 200,000 cells) from induced htNSCs (labelled as induced Ex/Mv) versus control htNSCs (labelled as Control Ex/Mv) for 2 d, and cells were lysed and examined for N1, N2, N3, S, and E gene segments along with sub-genomic E region (Sg-E) of SARS-CoV-2 using qPCR. **(B)** htNSC-PIWIL2 KO versus control htNSCs were subjected to twice exposures of F1-6 through transfection as described in Fig 5A. Same amount of Ex/Mv (3.5 μg per 200,000 cells) from these induced htNSC-PIWIL2 KO versus induced control htNSCs or vehicle were used to treat hACE2-A549 cells upon infection of MOI 1.0 SARS-CoV-2 for 2 d. Then, cells were lysed and examined for N1, N2, N3, S, and E gene segments along with sub-genomic E region (Sg-E) of SARS-CoV-2 using qPCR. Data of expression is represented as fold change with respect to control whose average was adjusted as 1. *P < 0.05, **P < 0.01, ***P < 0.001, ****P < 0.0001; ANOVA/post hoc was applied between the indicated groups, data reflect mean ± SEM.

Given that viral RNA fragments-stimulated htNSCs lead to Ex/Mv with higher levels of some piRNAs which could putatively target SARS-CoV-2, we then studied if this induction could help the antiviral action of htNSC Ex/Mv. To discern a possible extra antiviral effect by these induced Ex/Mv, high-dose infection at MOI 1.0 was used for cultured hACE2-A549 cells. The treatment was based on the same dose of induced htNSC Ex/Mv versus control htNSC Ex/Mv (3.5 μg Ex/Mv for 200,000 hACE2-A549 cells), whereas vehicle treatment was included to provide as a control. After 2-d infection and treatment, we collected these cells for determining viruses by qPCR measurement of viral genomic copies. We found that although treatment of htNSC Ex/Mv led to a significant effect against SARS-CoV-2, this antiviral effect was further enhanced through treatment with induced htNSC Ex/Mv (Fig 5A). Sub-genomic qPCR analysis revealed that compared to control htNSC Ex/Mv, induced htNSC Ex/Mv showed a stronger effect to reduce SARS-CoV-2 replication (Fig 5A). In summary, NSCs can be induced via viral RNA fragment stimulation to produce induced Ex/Mv with increase levels in some sequence-matched piRNAs for an enhanced antiviral effect in targeting SARS-CoV-2.

### Antiviral action of induced htNSC Ex/Mv requires PIWI-piRNA system

Finally, we studied if the PIWI-piRNA system could be important for the enhanced antiviral effect of induced htNSC Ex/Mv. To do so, htNSC-PIWIL2 KO versus control htNSCs were twice exposed to viral RNA fragments F1–6 for stimulation, as similarly described in Fig 4. Then, Ex/Mv were isolated and purified from htNSC-PIWIL2 KO versus control htNSCs, and the same dose of these Ex/Mv (3.5 μg Ex/Mv for 200,000 hACE2-A549 cells) was used to treat hACE2-A549 cells upon infection of MOI 1.0 SARS-CoV-2. After 2 d of incubation, these cells were harvested, lysed, and analyzed for genomic copies of SARS-CoV-2. As shown in Fig 5B, whereas induced htNSC Ex/Mv showed an enhanced antiviral effect as similarly observed in Fig 5A, ablation of PIWIL2 led to abrogation of not only the enhanced effect but also the basal effect of Ex/Mv against SARS-CoV-2. Sub-genomic qPCR assay further showed that replication of SARS-CoV-2 in hACE2-A549 cells greatly decreased by induced htNSC Ex/Mv but only weakly by induced htNSC-PIWIL2 KO Ex/Mv (Fig 5B). Taken together, the PIWIL2-piRNA system in NSC Ex/Mv is important for basal as well as RNA-stimulated antiviral actions of these extracellular particles.

## Discussion

In this report, we demonstrated that murine piRNAs-containing Ex/Mv from htNSCs can target against SARS-CoV-2 through innate immunity, and the antiviral effects of these extracellular vesicles can be further induced to increase through an adaptive immunity-like mechanism after twice exposure to viral RNA fragments. Further, loss-of-function experiments by ablating PIWIL2 revealed that the PIWI-piRNA system is required for both innate and induced antiviral actions of htNSC Ex/Mv. Overall, despite that this study was based on in vitro infection models, this work is potentially significant and informative, as it provides a novel working model and a new direction for exploring options to combat RNA viruses.

Murine NSCs have large capabilities of making Ex/Mv that are enriched with small RNAs (4). We recently found that piRNAs represent an important pool for NSC Ex/Mv small RNAs and that these piRNAs-containing NSC Ex/Mv can work to suppress the infection of several pseudotyped RNA viruses (2). In the current study, we targeted pandemic SARS-CoV-2 and showed a strong treatment effect by htNSC Ex/Mv against this virus. NSC-derived Ex\Mv are unlikely abundant in the blood, given the large volume of blood and also since NSC-derived Ex\Mv might not easily pass across the BBB. However, NSC-derived Ex\Mv are likely significant for the brain through the levels in the cerebrospinal fluid, supported by the endocrine feature of these extracellular vesicles that we recently revealed (3, 4). In conjunction with our recent findings based on pseudotyped viruses (2), the results in this work from using SARS-CoV-2 further confirm that NSC Ex/Mv have antiviral immunity functions. We reason that this feature of NSC Ex/Mv is important for protecting the brain from viral infection, especially because the brain is in anatomically separated from peripheral immune system due to the BBB. We also speculate that some peripheral cells could have some features of producing piRNA Ex/Mv, such as peripheral tissue stem cells, if there are close relationships between the PIWI-piRNA system and stem cells in general. Thus, identification of piRNA-related peripheral cells will be important, especially given that NSCs are more limited and more difficult to establish compared to peripheral cells.

In this study, we used sub-genomic assay for SARS-CoV-2 genome, a method that reported replication of viruses in cells (32). The data support that NSC Ex/Mv can suppress SARS-CoV-2 replication. Because this effect was abrogated when PIWIL2 was absent, we conclude that the PIWI-piRNA system is important for these antiviral effects. A limitation of this study was that piRNA sequences entirely relied on the information available in public depository while many of them have not been verified. What makes it more challenging is, defining piRNAs, perhaps in particular mammalian piRNAs, might not be clearly based on sequence information, for example, although piRNAs have strong bias for uridine at the five end and/or for adenosine at position 10, exceptions to these features are also reported (33, 35, 36). In this work, among piRNAs that we analyzed, some species fit with these features, whereas others do not, so future biochemical and functional assessment are anticipated to characterize these small RNA species. Whereas this study is to give a conceptual framework for connecting Ex/Mv piRNAs with antiviral immunity, it will be particularly meaningful to evaluate individual piRNAs or in certain combination in providing an antiviral action. Also, it is possible that some antiviral effects of Ex/Mv could be piRNA-independent, for instance, we did recently observe that NSC Ex/Mv can directly bind to and sequester pseudotyped viruses (2). Clearly future studies are needed to assess piRNA-independent versus piRNA-dependent antiviral immunity of NSC Ex/Mv.

In this work, we demonstrated results showing viral RNA fragments can stimulate NSCs to induce Ex/Mv with increased expression levels of piRNAs which could potentially target some of these RNA sequences. These findings imply that RNA vaccine strategy can be extended to comprise a small RNA-driven adaptive immunity that is independent of antibody or immune cells, given that viral RNA fragments in our experiments did not involve protein translation from them, and our in vitro model did not involve classical adaptive immune cells such as lymphocytes. Currently, RNA vaccines have been developed to effectively combat SARS-CoV-2, and the antiviral effects of these RNA vaccines are believed to rely on the production of neutralization antibody against SARS-CoV-2 spike protein. The findings in our study would call for an investigation regarding if a small RNA-dependent adaptive immune mechanism could be induced by these vaccines. If so, it would be interesting to discern the contributions from induced neutralization antibody versus induced piRNAs to the antiviral actions of these vaccines.

## Materials and Methods

### Cell models and culture

Primary culture of htNSCs was performed as described previously (1, 2, 3, 4). Hypothalamic tissues from newborn C57BL/6 mice were dissected and cut into pieces around 1 mm^3^ size and then digested using TrypLE Express (Gibco) at 37°C for 10 min; cells were centrifuged and resuspended in NSC medium composed of neurobasal-A (Gibco), 0.25% GlutaMAX supplement (Gibco), 2% B27 without vitamin A (Gibco), 10 ng ml^−1^ EGF (Gibco), 10 ng ml^−1^ bFGF (Gibco), and 0.5% penicillin–streptomycin (Gibco). After resuspension, cells were seeded in ultra-low attachment surface six-well plate (Corning). 1 wk later, neurospheres from culture were collected, trypsinized, passaged, and maintained as NSC culture. Procedures for animal use were approved by the Institutional Care and Use Committee of Albert Einstein College of Medicine (protocols # 00001111, 00001397, 00001398, 00001399). The model of htNSC-PIWIL2 KO was generated by subjecting htNSCs to mammalian CRISPR lentiviral infection followed by blasticidin selection and were then maintained as a stable htNSC cell line. The model of hACE2-A549 cells was generated through infecting A549 cells (CCL-185; ATCC) with hACE2-expressing lentiviruses for 2 d followed by puromycin selection leading to a stable cell line. A549 and hACE2-A549 cells were cultured using F-12K nutrient mixture (Gibco). HEK293T (CRL-3216; ATCC) and BHK21 (EH1011; Kerafast) cell cultures were prepared using Dulbecco’s modified Eagle medium (Gibco). HEK293T, BHK21, A549, and hACE2-A549 cell culture medium was supplemented with 5–10% heat-inactivated fetal bovine serum, FBS (Gibco), and 1% penicillin–streptomycin (Gibco). BHK21 cell culture was maintained at 37°C and 7% CO_2_ humidified atmosphere, and all other cell models were maintained at 37°C and 5% CO_2_ humidified atmosphere. 0.25% trypsin–EDTA (Gibco) was used for trypsinization of attached culture for passaging. Lipofectamine 3000 (Invitrogen) was used for cell transfection per the manufacturer’s instruction.

### Ex/Mv production and isolation

Neurosphere culture of htNSCs was used to obtain Ex/Mv that were released from these cells. Ex/Mv were isolated from htNSC culture medium through using differential centrifugation technique as described previously (2, 3, 4). Briefly, an exosome-free medium was used to culture htNSCs for up to 2 d, and the medium during various time intervals was harvested and centrifuged at 2,000*g* for 10 min to remove debris, followed by filtration through 0.8-μm-pore-size filter (Corning). The filtrates were then purified for Ex/Mv via ultracentrifugation at 110,000*g* at 4°C for 75 min, and purified Ex/Mv were resuspended in PBS. Fresh Ex/Mv particles were made within 2–3 wk before an experiment for its use, while repeated freezing and thawing were avoided. Ex/Mv purified by ultracentrifugation were quantified using the Pierce BCA protein assay kit (Thermo Fisher Scientific), a well-established approach for purified exosome quantification (37, 38, 39). In brief, aliquots of samples were lysed on ice using 1× RIPA buffer followed by centrifugation at 12,000*g* for 15 min at 4 degrees. Ex/Mv protein lysate and standard protein samples were incubated with BCA reagent for 30 min at 37 degree, and absorbance was read at 562 nm using SpectraMax iD3 spectrophotometer. The obtained values were plotted with reference to standard plot and protein concentration of Ex/Mv was calculated. The BCA assay was performed using the Pierce BCA protein assay kit (Thermo Fisher Scientific).

### Plasmids and recombinant lentiviruses

Expression plasmids pCAGGS containing SARS-CoV-2 (Wuhan-Hu-1) spike glycoprotein (NR-52310) was obtained from BEI resources. Vector pCAGGS containing SARS-CoV-1 spike glycoprotein was gifted by Whittaker Lab, Robert Frederick Smith School of Chemical & Biomolecular Engineering, Cornell University. Lentiviral plasmid vector EF1A promoter–driven hACE2 (VB200000-2751mcf) and mammalian CRISPR lentiviral plasmid vector for PIWIL2 knockout (VB201202-1202gqa) were purchased from Vector Builder. Lentiviruses were produced by co-transfection of cultured HEK293T cells by lentiviral and packaging plasmids, purified via ultracentrifugation and then quantitated with Lenti-X GoStix Plus Kit (TaKaRa), as similarly described in previous publications (1, 2, 3, 4, 40).

### SARS-CoV-2 and cell treatment

SARS-CoV-2 viruses (USA-WA1/2020, NR-52281; BEI Resources) were generated as previously described (28). Briefly, Vero E6 monolayer was infected with serial dilutions of viruses and then cytopathic effects for these cells were scored. The dilution at which half of cells showed cytopathic effects was calculated using Reed and Muench method as TCID50 per ml. This was used to calculate MOI values for further infections. For testing the effect of htNSC Ex/Mv, SARS-CoV-2 was added to about 80% confluent hACE2-A549 cells to achieve the final indicated MOI, and these cells were simultaneously treated with a dose of Ex/Mv or vehicle control. Cells were maintained in these conditions for 2 d before they were treated with Trizol for RNA analysis. All these procedures were performed in Columbia Aaron Diamond AIDS Center BSL3 facility per the guidelines of the high containment BSL3 laboratory for Columbia University.

### Pseudotyped SARS-CoV and cell treatment

Pseudotyped ΔG-luciferase rVSV with SARS-CoV-2 glycoprotein or SARS-CoV-1 glycoprotein was performed as similarly described in recent publication (2). In brief, 80% confluent BHK21 were transfected with SARS-CoV-2 or SARS-CoV-1 spike glycoprotein expression plasmid. Transfected cells were culture at 37°C with 7% CO_2_ in reduced serum DMEM for 30 h. Next day, the transfection medium was removed, and cells were infected with pseudotyped ΔG-luciferase rVSV in serum and antibiotic free DMEM at an MOI of 3. After 4 h of infection, the cells were washed with 1× DPBS, reduced serum DMEM was added, and cells were further cultured for another 24 h. On the following day, pseudotyped viruses in the culture medium were collected and filtered using 0.45-μm-pore-size filter (Corning); the filtrate was ultracentrifuged with 20% sucrose cushion at 100,000*g* for 35 min at 4°C and reconstituted in PBS for experimental use. Cultured hACE2-A549 cells at an appropriate density were added in the medium with a dose of pseudotyped SARS-CoV-2 or pseudotyped SARS-CoV-1 and about 1 h later were added with a dose of Ex/Mv or vehicle in the medium. Cells were maintained under these conditions for 3 d before fixed or lysed for subsequent assays.

### RNA in vitro transcription and cell transfection

DNA products of different SARS-CoV-2 genomic fragments were generated by the method of PCR using the cDNAs of SARS-CoV-2 (USA-WA1/2020) viral genome as the template and specific primers listed in Table S1. These PCR products were each cloned into pCR-Blunt II-TOPO vector with T7 promoter using Zero Blunt TOPO PCR Cloning Kit (Invitrogen). Restriction enzyme digestion was carried out to screen the correct orientation of each PCR fragment containing T7 promoter. The clones containing each PCR product were linearized for in vitro transcription followed by poly (A) tailing with HiScribe T7 ARCA mRNA Kit (New England Biolabs). The resulting RNA fragments were purified and quantitated for the use of cell transfection.

Table S1 Primer information in this study.

### RNA assays by qPCR

Total RNA from cells infected with SARS-CoV-2 were extracted and reverse transcribed to cDNA. Real-time qPCR assays for SARS-CoV-2 genomic sequences in N1, N2, N3, S, and E region as well as sub-genomic E region (sg-E) were performed using power-up SYBR green master mix (Thermo Fisher Scientific) (41, 42, 43). All these qPCR results were normalized using the expression of house-keeping β-actin as an internal control. Primers used for these assays are listed in Table S1. Total small RNAs from purified Ex/Mv were isolated using Ambion mirVana miRNA isolation kit (AM1560). The concentration and purity of total small RNAs were measured at 260 and 280 nm absorbance. Extracted small RNAs were polyadenylated by Lucigen poly(A) polymerase tailing kit (PAP5104H) and reverse transcribed by SuperScript III Fist-Strand Synthesis System (18080-051; Invitrogen) with universal RT primer to produce cDNAs for real-time qPCR via SYBR Green PCR Master Mix (4309155; Applied Biosystems). All piRNA qPCR results were normalized according to house-keeping U6 levels which were stable among different experimental conditions. Specific primers and the universal reverse primer used for piRNA qPCR assays are listed in Table S1. ACE2 mRNA levels in A549 and hACE2-A549 cells was evaluated by subjecting total RNAs isolated from these cells to qPCR, and these qPCR results were also normalized via the expression levels of house-keeping β-actin as an internal control.

### Western blotting

Cells were lysed on ice using RIPA buffer (Alfa Aesar) with protease inhibitor cocktail (Thermo Fisher Scientific). After protein quantification, equal quantities of cell lysate samples were mixed with Laemmli buffer (Pierce) and boiled at 95°C for 5 min. Samples were run on SDS–PAGE gel and were transferred onto a 0.2-μm-PVDF membrane (Bio-Rad). The membranes were blocked using 5% non-fat milk in TBST and were blotted with anti-ACE2 rabbit mAb (SN0754; Invitrogen), anti-PIWIL2 mouse mAb (sc-377258; Santa Cruz Biotechnology), anti-GAPDH mouse mAb (GA1R; Invitrogen), or anti–β-actin rabbit pAb (4967S; Cell Signaling Technology) overnight at 4°C in 5% non-fat milk in TBST. Secondary HRP-conjugated antibodies subsequently applied including antimouse (7076P2; Cell Signaling Technology) or anti-rabbit (7074P2; Cell Signaling Technology). The membranes were probed using the ECL system (Bio-Rad) and images were developed on the Image Studio software.

### Immunostaining

Cells were seeded on chamber slides (Nunc Lab-Tek), after reaching 70% confluency the cells were fixed using 4% PFA for 15 min at room temperature. Neurospheres were fixed in 4% PFA for 30 min at room temperature, embedded in OCT compound (Scigen), and cryosectioned for immunostaining. Fixed cells or neurosphere sections were washed with PBS three times and incubated with permeabilization buffer for 15 min (0.1% Triton-X). Cells were incubated overnight at 4°C with primary antibodies anti-ACE2 rabbit mAb (SN0754; Invitrogen), anti-PIWIL2 mouse mAb (sc-377258; Santa Cruz Biotechnology), or anti-luciferase, firefly rabbit pAb (AB3256; EMD Milipore Corp) in 5% BSA. Subsequently, after three times of wash, secondary antibodies applied including anti-rabbit or anti-mouse conjugated with Alexa Fluor 633 or 555 with 5% BSA for 1 h at room temperature. Finally, after washing three times, Vecta-Shield mounting medium containing DAPI was mounted, and images were captured using Leica SP8 confocal microscope. Images were developed using FIJI software.

### Biochemical assays

Firefly luciferase activities in cultured cells were analyzed through applying equal amount of protein lysate to One-glo Luciferase assay system (Promega). The light emissions were measured using SpectraMax iD3. Small RNA isolated from purified Ex/Mv were quantified via spectrophotometer and subjected to small RNA chip assay with 2100 Agilent Bioanalyzer (Agilent technologies) for analysis of small RNA species (via the Molecular Pathology Platform, Herbert Irving Comprehensive Cancer Center, Columbia University).

### Statistical analysis

Sample sizes of each experiment were determined based on the relevant literature and our published as well as pilot studies. All experiments were repeated independently at least once or through complementary experiments. Experimental data were subjected to test for parametric distribution. All data are presented as mean ± SEM. Two-tailed unpaired *t* test was used for comparing data of only two groups, and one-way ANOVA and appropriate post hoc tests was used for comparing more than two groups of data. Prism software and Excel were used for statistical analysis and *P*-value of less than 0.05 was defined to be statistically significant.

## Data Availability

All data are available from the corresponding author upon request.

## Supplementary Material

Reviewer comments
