# Supplementary material for: Exosomes/microvesicles target SARS-CoV-2 via innate and RNA-induced immunity with PIWI-piRNA system
Source: Life Sci Alliance. 2021 Dec 3;5(3):e202101240. doi: 10.26508/lsa.202101240 (PMC8645330; doi:10.26508/lsa.202101240)
Supplement: Supplementary file 1 [file LSA-2021-01240_TableS1.docx]

**Supplemental Table 1. Primer information in this study.**

| **Primers for generating SARS-CoV-2 RNA fragments** | | | | | | | |
| --- | --- | --- | --- | --- | --- | --- | --- |
| **Fragment** | | | **Primer** | **Primer sequence** | | | |
| F1 | | | Forward | TATGTACACACCGCATACAG | | | |
|  | | | Reverse | CGCGGGTGATAAACATGTTA | | | |
| F2 | | | Forward | ACCCAGGAGTCAAATGGAAA | | | |
|  | | | Reverse | ACTGACTAGAGACTAGTGGC | | | |
| F3 | | | Forward | GAGGCTGGATTTTTGGTACT | | | |
|  | | | Reverse | CATGAATAGCAACAGGGACT | | | |
| F4 | | | Forward | AATAGGGGCTGAACATGTCA | | | |
|  | | | Reverse | ATCTGAAGGAGTAGCATCCT | | | |
| F5 | | | Forward | AAAGAGATGGCAACTAGCAC | | | |
|  | | | Reverse | AGGACAAGCAAAAGCAAATT | | | |
| F6 | | | Forward | AACACTTTGCTTCACACTCAA | | | |
|  | | | Reverse | CACATGGGGATAGCACTACT | | | |
| **Primers for piRNAs** | | | | | | | |
| **piRNA ID (piRNAQuest)** | | **Primer sequence** | | | | | **Label** |
| mmu_piRNA_553635 | | AACAAGTGAAGAAGAGCAAGAAGGA | | | | | O1-2 |
| mmu_piRNA_443751 | | TATTTAAACTGTCTTATGTGTCTCCA | | | | | O1-3 |
| mmu_piRNA_923720 | | AACACGCTTTCCAGAGTTGTTGTAC | | | | | O1-7 |
| mmu_piRNA_555154 | | AACAAGTGAAGACCCAGTCCCTAC | | | | | S1 |
| mmu_piRNA_801725 | | AACAAGTGTGAAGGTGTCTTTGTCA | | | | | S2 |
| mmu_piRNA_562740 | | AACAAGTGAAGTCTGCCTGTGAAG | | | | | S3 |
| mmu_piRNA_498273 | | AACACGCTCCTGAAGAAGAATCAC | | | | | S4 |
| mmu_piRNA_233057 | | AACAAGGTGAAGTCTGCCTGTGA | | | | | S5 |
| mmu_piRNA_858506 | | AACAAGTTCAAGGCCAGCAGC | | | | | O3-1 |
| mmu_piRNA_561925 | | AAGCGCCTTGAAGTAACTGTGTATA | | | | | O3-2 |
| mmu_piRNA_443463 | | AACACGCTATTGTGTGAATTTGGTT | | | | | O3-3 |
| mmu_piRNA_443462 | | TATTGTGTGAATTTGGTTTTGTCATT | | | | | O3-4 |
| mmu_piRNA_443460 | | TATTGTGTGAATTTGGTTTTGTCAGG | | | | | O3-5 |
| mmu_piRNA_88635 | | ATTGTGTGAATTTGGTTTTGTCCTGG | | | | | O3-6 |
| mmu_piRNA_88634 | | ATTGTGTGAATTTGGTTTTGTCATGG | | | | | O3-7 |
| mmu_piRNA_88633 | | ATTGTGTGAATTTGGTTGTGTCATGG | | | | | O3-8 |
| mmu_piRNA_86326 | | AACAAGATGTTCTTCAGGCTCCC | | | | | O3-9 |
| mmu_piRNA_104250 | | AACACGCCAGAAGATCAGGAACTAA | | | | | E1 |
| mmu_piRNA_181390 | | AAGCGACCGAAGGTTTTACAAGATA | | | | | E2 |
| mmu_piRNA_466370 | | TCAGGACCTCTAGAAGAACAATCAGT | | | | | E4 |
| mmu_piRNA_419319 | | AACGGCTAGTTTTTCTGTTCAATGG | | | | | G1 |
| mmu_piRNA_419318 | | ACGCCGTAGTTTTTCTGTTAAGTGA | | | | | G2 |
| mmu_piRNA_865863 | | AACACGCTTCCAAACAGAAAATGC | | | | | G3 |
| mmu_piRNA_475430 | | AACACGCTCCAAACAGAAGAACTAG | | | | | G4 |
| mmu_piRNA_475429 | | AACACGCTCCAAACAGAAAAGCTTA | | | | | G5 |
| mmu_piRNA_700126 | | AACAAGTGCTTCTTTCAGACTTCCC | | | | | M1 |
| mmu_piRNA_379382 | | AACAAGTAGCAATTCCACCGGTG | | | | | M2 |
| mmu_piRNA_167046 | | AACAAGCTGTACAAGCAAAGCTCTT | | | | | M3 |
| mmu_piRNA_142622 | | AACAAGCCTGTATGCAGCAAAATG | | | | | M4 |
| mmu_piRNA_432244 | | AACACGCTATGAGGACTTTGAAAGT | | | | | O6-1 |
| mmu_piRNA_25718 | | AAGCGACCAATTTGCTTTTGCTTTA | | | | | O7-1 |
| mmu_piRNA_867226 | | AACAAGTTCCAGAAGAGCCAGGT | | | | | O7-2 |
| mmu_piRNA_320536 | | AACAAGTACACTCTTGGTAGTGGGG | | | | | O7-3 |
| mmu_piRNA_934618 | | AACAAGTTTTAGCCTTTCTGCCGTT | | | | | G6 |
| mmu_piRNA_292200 | | AACACGCTAAGGAATAGCAGAATGC | | | | | G7 |
| mmu_piRNA_934618 | | AACAAGTTTTAGCCTTTCTGCCGTT | | | | | O7-4 |
| mmu_piRNA_292200 | | AACACGCTAAGGAATAGCAGAATGC | | | | | O7-5 |
| mmu_piRNA_658285 | | AACAAGTGCAGCTACAGTTGTGTG | | | | | O8-1 |
| mmu_piRNA_718264 | | AACAATTGGACTTCCCTATGGTCGT | | | | | N1 |
| mmu_piRNA_153125 | | AACAAGCTCCATGAGCAGTGC | | | | | N2 |
| mmu_piRNA_288153 | | ACCACCGTAAGATGGTATTTCTAGC | | | | | N3 |
| mmu_piRNA_312079 | | AACACGCTAATTTCCTTGGGTTTGT | | | | | N4 |
| mmu_piRNA_657627 | | AACACGCTGCAGCAGATTTCTTATT | | | | | N5 |
| mmu_piRNA_521705 | | AACAAGTCTGCAGCAGGAAGAG | | | | | N6 |
| mmu_piRNA_647225 | | AACAAGTGCAAACCACACAAGG | | | | | G8 |
| mmu_piRNA_679242 | | AACAAGTGCCTTGTGTGGTGAAG | | | | | G9 |
| mmu_piRNA_865467 | | AACACGCTTCATTCTGCACAATGT | | | | | O10-1 |
| mmu_piRNA_811305 | | AACACGCTGTGCTATGTAGTTCTG | | | | | O10-2 |
| mmu_piRNA_402005 | | AACAAGTAGGGAGAGCTGCCC | | | | | U6 |
| mmu_piRNA_43308 | | AACAAGAGAAAAAGTGGTGGCTCTT | | | | | U7 |
| RT universal primer | | CAGTGCAGGGTCCGAGGTCAGAGCCACCTGGGCAATTTTTTTTTTTVN | | | | |  |
| PCR general reverse primer | | CAGTGCAGGGTCCGAGGT | | | | |  |
| U6 forward primer | | CTCGCTTCGGCAGCAC | | | | |  |
| U6 reverse primer | | AACGCTTCACGAATTTGCGT | | | | |  |
| **Primers for SARS-CoV-2 genomic regions** | | | | | | | |
| **Target viral gene** | **Primer** | | | | **Primer sequence** | | |
| N1 | Forward | | | | GACCCCAAAATCAGCGAAAT | | |
|  | Reverse | | | | TCTGGTTACTGCCAGTTGAATCTG | | |
| N2 | Forward | | | | TTACAAACATTGGCCGCAAA | | |
|  | Reverse | | | | GCGCGACATTCCGAAGAA | | |
| N3 | Forward | | | | GGGAGCCTTGAATACACCAAAA | | |
|  | Reverse | | | | TGTAGCACGATTGCAGCATTG | | |
| S | Forward | | | | ACAGGCACAGGTGTTCTTAC | | |
|  | Reverse | | | | GATCACGGACAGCATCAGTAG | | |
| E | Forward | | | | ACAGGTACGTTAATAGTTAATAGCGT | | |
|  | Reverse | | | | ATATTGCAGCAGTACGCACACA | | |
| Sg-E | Forward | | | | CGATCTCTTGTAGATCTGTTCTC | | |
| β-actin | Forward | | | | CACCATTGGCAATGAGCGGTT | | |
|  | Reverse | | | | AGGTCTTTGCGGATGTCCACGT | | |
| **Primers for ACE2 mRNA** | | | | | | | |
|  | Forward | | | | | TCCATTGGTCTTCTGTCACCCG | |
|  | Reverse | | | | | AGACCATCCACCTCCACTTCTC | |
